# Photodriven water oxidation initiated by a surface bound chromophore-donor-catalyst assembly†

**DOI:** 10.1039/d1sc03896f

**Published:** 2021-10-11

**Authors:** Degao Wang, Zihao Xu, Matthew V. Sheridan, Javier J. Concepcion, Fei Li, Tianquan Lian, Thomas J. Meyer

**Affiliations:** Engineering Laboratory of Advanced Energy Materials, Ningbo Institute of Industrial Technology, Chinese Academy of Sciences Ningbo Zhejiang 315201 China wangdegao@nimte.ac.cn; Qianwan Institute of CNiTECH Zhongchuangyi Road, Hangzhou Bay District Ningbo Zhejiang 315336 China; Department of Chemistry, University of North Carolina at Chapel Hill Chapel Hill NC 27599 USA; Department of Chemistry, Emory University Atlanta GA 30322 USA; Chemistry Division, Brookhaven National Laboratory Upton New York 11973 USA; State Key Laboratory of Fine Chemicals, Dalian University of Technology Dalian 116024 China

## Abstract

In photosynthesis, solar energy is used to produce solar fuels in the form of new chemical bonds. A critical step to mimic photosystem II (PS II), a key protein in nature's photosynthesis, for artificial photosynthesis is designing devices for efficient light-driven water oxidation. Here, we describe a single molecular assembly electrode that duplicates the key components of PSII. It consists of a polypyridyl light absorber, chemically linked to an intermediate electron donor, with a molecular-based water oxidation catalyst on a SnO_2_/TiO_2_ core/shell electrode. The synthetic device mimics PSII in achieving sustained, light-driven water oxidation catalysis. It highlights the value of the tyrosine–histidine pair in PSII in achieving efficient water oxidation catalysis in artificial photosynthetic devices.

## Abstract 2

We describe a single molecular assembly electrode that mimics PSII. Flash photolysis revealed the electron transfer steps between chromophore light absorption and the creation and storage of redox equivalents in the catalyst for water oxidation.

## Graphical abstract

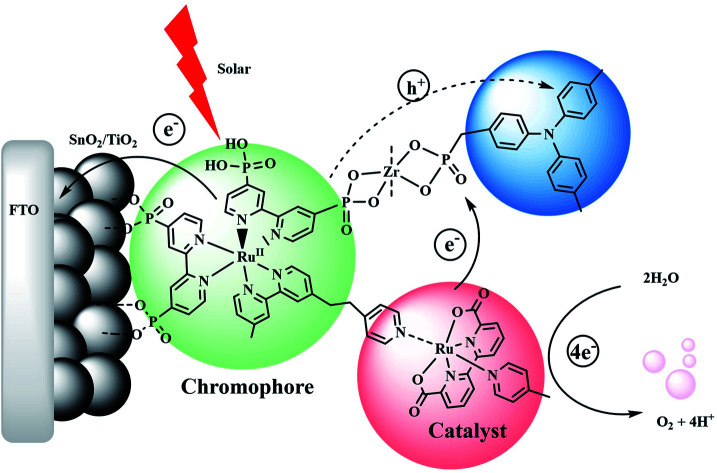

## Introduction

A central goal in artificial photosynthesis is storing solar energy from sunlight in chemical bonds.^1–5^ Fujishima and Honda first demonstrated that direct band gap excitation of the semiconductor, TiO_2_, led to water photolysis and a pathway for solar energy conversion based on water splitting (2H_2_O → O_2_ + 2H_2_).^6^ The use of semiconductor electrodes has continued to evolve with progress made in improving light absorption, charge separation, charge transport, and catalysis rates at semiconductor surfaces.^7–13^ The latter includes the development of dye-sensitized photoelectrosynthesis cells (DSPECs) that integrate separate semiconductor electrodes with molecular complexes for light absorption and catalysis.^14–25^ With this approach, each component in a DSPEC can be investigated separately, finely tuned to maximize performance, and then integrated with a semiconductor(s) in an appropriate surface architecture.

**Scheme 1:**
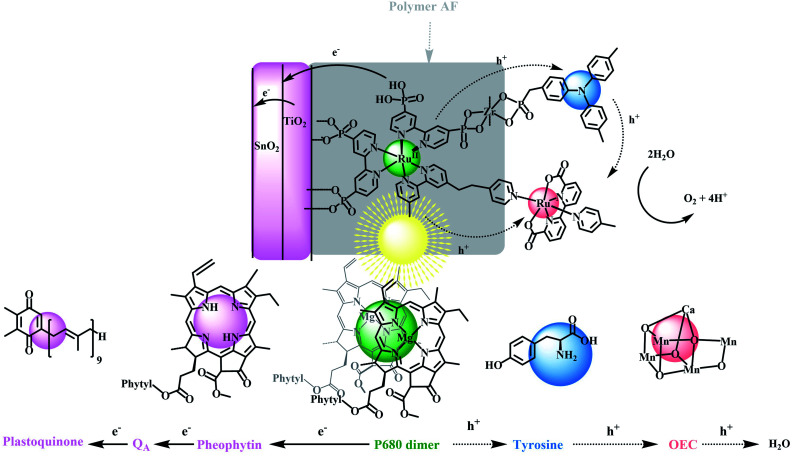
(Top) Structure and direction of electron flow in the dye sensitized photoelectrosynthesis cell (DSPEC), FTO**|SnO2/TiO2|–RuIIP(TPA)(Cat)2+**. The structure omits the external, stabilizing polymer, 4,5-difluoro-2,2-bis(trifluoromethyl)1,3-dioxole (**AF**). In the final assembly, **Cat** is a derivative of a Ru(ii)-2,2′-bipyridine-6,6-dicarboxylate based catalyst for water oxidation and **TPA** is a derivative of tri-phenyl amine. (Bottom) Illustrating the related components in PSII with arrows indicating the direction of light-driven electron transfer following excitation of the external chromophore.

DSPEC cells typically utilize chromophores and catalysts that readily attach to oxide surfaces, have high light absorption and strong oxidizing potentials for driving water oxidation at molecular catalysts.^19,20,26^ Although significant progress has been made in this area, especially with the development of ultra-fast catalysts by Sun and co-workers, stabilization of DSPEC devices may present the most significant current bottleneck in practical applications. In Nature, the photosynthetic reaction center evolved over millions of years with water oxidation occurring in the photosystem II (PSII) protein. This protein is responsible for light-driven water oxidation (2H_2_O → O_2_ + 4H^+^ + 4e^−^) in nature.^1,27–30^ Mimicking the natural system is an inspiration for chemists but given, its high molecular weight and structural complexity, has presented major challenges.^22,31–33^ PSII is a complex molecular assembly, but its basic components are a spatially extended, light-absorbing chlorophyll array, a P680 chlorophyll acceptor, a tyrosine mediator, and an oxygen-evolving catalyst (OEC), as illustrated in Scheme 1.^34–40^

The relative simplicity of the molecular photoelectrochemical approach described here is notable for achieving many of the key components of PSII. A chromophore, bound to a semiconductor surface, is excited to create a molecular excited state. The excited state then undergoes electron transfer to a SnO_2_/TiO_2_ semiconductor electrode, with an internal core/shell structure that facilitates electron transfer to a photocathode for water reduction.^41–44^ The oxidative equivalents from the chromophore undergo intra-assembly electron transfer to a water oxidation catalyst either directly or *via* a mediator where water oxidation finally occurs. Because of their relatively high visible absorptivity, and high stabilities in aqueous solutions, polypyridyl Ru(ii) complexes have been used as the light-absorbing chromophores (**Chrom**) in the preparation of these types of assemblies where they are co-loaded with, or chemically linked to catalysts (**Cat**) for water oxidation.

The reaction sequence for water oxidation is illustrated in eqn (1)–(3). It is based on a chromophore–catalyst assembly formed on a SnO_2_/TiO_2_ core/shell electrode. In the reaction sequence for water oxidation, the surface-bound chromophore is excited and undergoes electron transfer to TiO_2_ followed by electron transfer to an inner SnO_2_ layer driven by the lower conduction band of SnO_2_ compared to TiO_2_. Electrons removed from SnO_2_ at the back contact produce the photocurrent that is transferred to an external cathode for proton or CO_2_ reduction.^45–48^

1 SnO_2_/TiO_2_|Chrom,Cat + *hν* → SnO_2_/TiO_2_(e^−^)|Chrom^+^,Cat, *excitation and injection*

2 SnO_2_/TiO_2_(e^−^)|Chrom^+^,Cat → SnO_2_(e^−^)/TiO_2_|Chrom,Cat^+^, *intra-film electron transfer*

3 SnO_2_(e^−^)/TiO_2_|Chrom,Cat^+^ − e^−^ → SnO_2_/TiO_2_|Chrom,Cat^+^, *photocurrent*

In PSII, a sequence of multi-step electron transfers controls the kinetics and balances the oxidation–reduction reactions.^49^ The four underlying redox reactions leading to water oxidation all occur on the millisecond timescale.^50–53^ In comparing the surface activation cycle in eqn (1)–(3) with PSII, a missing component in many artificial photosynthesis devices is the addition of a mediator that mimics the tyrosine–histidine redox couple in PSII.^21,54^ In the analogous reactions in eqn (4)–(6) for the DSPEC phoroanode an additional redox couple (**Donor**) added to the DSPEC plays a key role mediating electron transfer between the oxidized chromophore and catalyst which mimicks the role of tyrosine as an electron transfer mediator in PSII.^32,55^

4 SnO_2_/TiO_2_|Chrom,Donor,Cat + *hν* → SnO_2_/TiO_2_(e^−^)|Chrom,Donor^+^,Cat

5 SnO_2_/TiO_2_(e^−^)|Chrom,Donor^+^,Cat → SnO_2_(e^−^)/TiO_2_|Chrom,Donor,Cat^+^

6 SnO_2_(e^−^)/TiO_2_|Chrom,Donor,Cat^+^ − e^−^ → SnO_2_/TiO_2_|Chrom,Donor,Cat^+^

In PSII, tyrosine inhibits back electron transfer from the oxidized catalyst and stabilizes the assembly by storing transient oxidative equivalents near the catalyst. In addition the proton-coupled electron transfer (PCET) reaction at the tyrosine–histidine which influences the oxidizing power of the redox couple, also plays a role in the dynamics of charge separation and alters the hydrogen bonding environment near the active site of the catalyst.^56^ Nevertheless, in the model here the primary focus is the role as a one-electron transfer mediator. In filling this role in the molecular model, the redox potential for a mediating couple should fall between the ground-state potential for the chromophore and the redox couple(s) of catalyst associated with the rate-limiting step in water oxidation catalysis. Intervention of the mediator, therefore, may occur in any or all of the four photoactivation steps typically associated with the 4e^−^ oxidation of water. It is also desired that the different redox states of the mediator be optically transparent in the visible and have good stability in both redox states in aqueous solutions. Triphenylamine (TPA) was chosen here because it meets many of these desired properties.

In mimicking PSII, we describe here a chemical approach based on the reactions in eqn (4)–(6). It utilizes a semiconductor-surface assembly that mimics PSII by adding an electron transfer mimic for tyrosine to complete the PSII model. In the final electrode, an external ∼5 nm thick TiO_2_ shell was used as an external layer on an internal SnO_2_ core on a fluorine-doped tin oxide (FTO) electrode. A derivative of the polypyridyl Ru(ii) complex, [Ru(bpy)_3_]^2+^, with bpy = 2,2′-bipyridine, was used as the light absorber with a triphenylamine derivative as the electron transfer donor and mediator.^57,58^ The catalyst for water oxidation was a derivative of the Ru(ii)-2,2′-bipyridine-6,6-dicarboxylate based, Ru(bda)(py)_2_ (py, pyridine), first described by Sun and co-workers, and, as mentioned above, notable for their rapid rates of oxygen evolution.^59–63^ The final assembly was stabilized by adding a 1–2 nm overlayer of the fluorinated DuPont AF polymer, 4,5-difluoro-2,2-bis(trifluoromethyl)-1,3-dioxole, which creates an external hydrophobic environment with the structure shown in Fig. S1.†^64^

As shown in Scheme 1, in the final electrode assembly, FTO**|SnO2/TiO2|–RuIIP(TPA)(Cat)2+|AF**, the key elements of PSII are included in a working photoelectrode for water oxidation. In a 0.1 M phosphonate buffer solution at pH 7 in 0.4 M NaClO_4_, with an applied bias of 0.6 V *vs.* NHE, the electrode produced O_2_ with an efficiency of 83% and an IPCE value of 10.9% at its absorption maximum of 460 nm.

## Results and discussion

### Film characterization

Mesoporous films of nanoITO, TiO_2_, ZrO_2_ and SnO_2_/TiO_2_, for spectral and electrochemical measurements, were prepared by known literature procedures.^38,65^ In brief, a TiO_2_ paste, prepared by using a known literature procedure, was deposited on FTO glass with a sheet resistance of 15 Ω sq^−1^ by using a doctor blading method on a layer of Scotch tape. Following a heat treatment, films of 4 micron and 20 nm TiO_2_ nanoparticle films were produced.^66^ Four micron, 20 nm particle ITO films utilized the same procedure but with different paste compositions, as reported in the literature.^67–69^ A nanoSnO_2_ paste, and films with core–shell SnO_2_/TiO_2_ (4 micron, 20 nm) structures, were coated with ∼4.5 nm TiO_2_ layers by using atomic layer deposition, Fig. S2 and S3.†^70–74^

### Absorption spectra

**Fig. 1:**
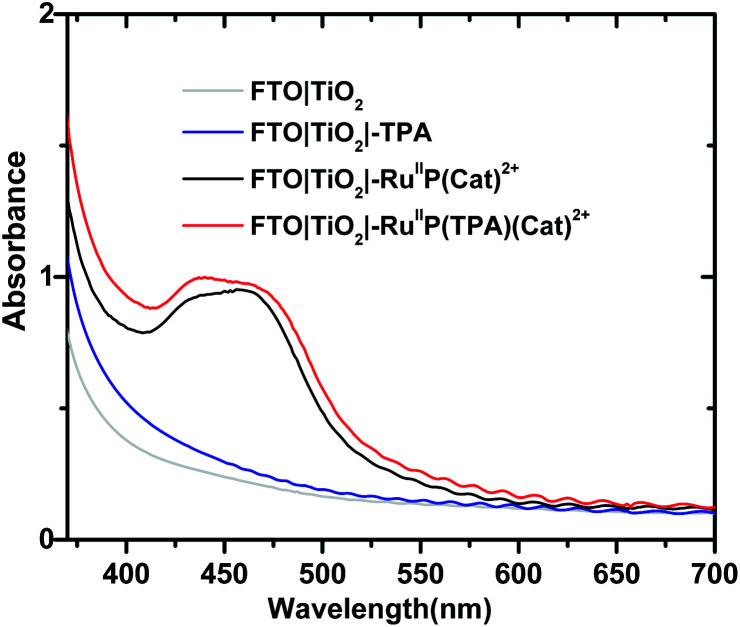
Absorption spectra for FTO**|TiO2|–RuIIP(Cat)2+**, before (black), and after addition of **TPA** to give FTO**|TiO2|–RuIIP(TPA)(Cat)2+**. Spectra of the related electrodes, FTO**|TiO2** (gray) and FTO**|TiO2|-TPA**, after TPA deposition (blue) are also shown. The spectra were obtained at room temperature in air.

Formation of assemblies on oxide surfaces was monitored by UV-visible measurements in air. Results are shown in Fig. 1 for films of FTO**|TiO2|-TPA**, FTO**|TiO2|–RuIIP(Cat)2+**, FTO**|TiO2|–RuIIP(TPA)(Cat)2+**, and the electrode substrate, FTO**|TiO2**. The absorption spectrum for FTO**|TiO2|-RuIIP2+** includes the expected metal-to-ligand charge-transfer (MLCT) absorption maximum at 460 nm, Fig. S4.†^39,75^ Addition of the catalyst to give the assembly, FTO**|TiO2|–RuIIP(Cat)2+**, results in additional features in the spectrum from the catalyst.^38,65^

The extent of surface loading, *Γ*, was determined by absorption measurements with, *Γ* = *A*/(*ε* × 1000), and *A* the absorbance at the wavelength of interest, *ε* is the molar extinction coefficient, and *Γ* is the surface coverage in mol cm^−2^. Following the surface loading procedures described in the Experimental, surface loading of the molecular was, *Γ* = 5 × 10^−8^ mol cm^−2^, based on measurements at 460 nm of chromophore with *ε*(460 nm) = 1.60 × 10^4^ M^−1^ cm^−1^ and catalyst absorptivity at 460 nm of 0.55 × 10^4^ M^−1^ cm^−1^.^38,39,65^ The loading level was comparable to surface loading levels for fully loaded surfaces of TiO_2_|–**RuIIP2+**.^76,77^ As expected, addition of the **TPA** electron transfer donor to the assembly to give, FTO**|TiO2|–RuIIP(TPA)(Cat)2+**, resulted in no significant change in the visible spectrum but with evidence for the added donor in the UV, Fig. 1.^57,58^

### Electrochemistry

Aqueous solution cyclic voltammograms were obtained for the derivatized electrodes at pH 7.0 in 0.1 M phosphonate buffers, in 0.4 M in NaClO_4_ on fully loaded planar FTO glass electrodes using a Ag/AgCl (3 M NaCl) as the reference electrode, Fig. S5–S7.† For the electrode FTO**|–RuIIP2+**, a reversible wave appeared for the Ru(iii/ii) couple at *E*_1/2_(**RuIII/II**) = 1.35 V *vs.* NHE at a scan rate of 50 mV s^−1^. For FTO|**-TPA**, the **TPA/TPA+˙** couple appeared at 1.08 V *vs.* NHE.

For the catalyst couples in the assembly, FTO**|–RuIIP(Cat)2+**, voltammograms at pH 7 are pH dependent, as they are for model complex Ru(bda)(py)_2_. Oxidation from Ru(ii) to Ru(iii) occurs with proton loss at a bound aquo ligand to give Ru^III^–OH^2+^ (**Cat′**) at *E*_1/2_ = 0.7 V; further oxidation to Ru^IV^=OH^2+^ (**Cat′′**) then occurs at 0.9 V.^65,78^ These oxidations are followed by a pH-dependent oxidation of Ru(iv) to Ru(v) at ∼1.0 V to form Ru^V^ = O (**Cat′′′**).^78^ The latter triggers water oxidation giving O_2_ with regeneration of the catalyst.^63,79^ Water oxidation occurs through an unstable, peroxo-bridged intermediate which decomposes and releases O_2_.^61,80,81^

### X-ray photoelectron spectroscopy (XPS)

To further confirm the characterization of the final assembly on metal oxide surfaces, X-ray photoelectron spectroscopy (XPS) was used to investigate interfacial elemental compositions for the surface-based structures. Based on the data shown in Fig. S8,† the Ru/P ratio was 0.55 in FTO**|SnO2/TiO2|–RuIIP(Cat)2+** and 0.32 in FTO**|SnO2/TiO2|–RuIIP(TPA)(Cat)2+**. Both were consistent with the proposed compositions of the final assemblies.

### Photostability

**Fig. 2:**
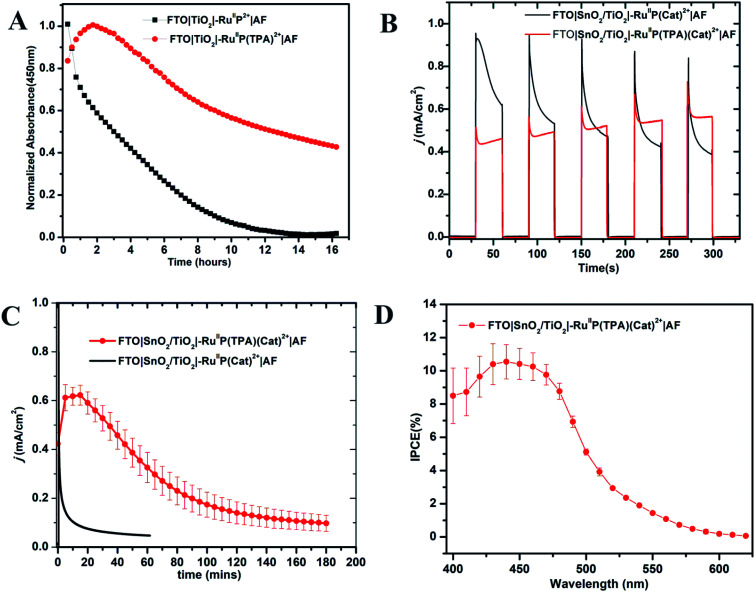
(A) Variations in surface coverage with time for the electrodes, FTO**|TiO2|–RuIIP2+**|**AF** and FTO**|TiO2|–RuIIP(TPA)2+**|**AF** with 455 nm, 475 mW cm^−2^ photolysis over 16 h periods in aqueous, pH 7, 0.1 M phosphonate buffers in 0.4 M NaClO_4_. Loss from the surface was monitored by following absorbance changes at 450 nm for the chromophore with corrections for light scattering by TiO_2_. (B) Current density–time (*j*–*t*) traces over 150 s dark–light cycles for water oxidation by FTO**|SnO2/TiO2|–RuIIP(Cat)2+**|**AF** (black) and FTO**|SnO2/TiO2|–RuIIP(TPA)(Cat)2+**|**AF** (red) at an applied bias of 0.6 V *vs.* NHE in 0.1 M phosphonate buffers in 0.4 M NaClO_4_ at pH 7.0. (C) A 3 h photoelectrochemical water oxidation cycle for FTO**|SnO2/TiO2|–RuIIP(TPA)(Cat)2+**|**AF** illuminated under the same conditions as in (b) compared to an electrode without the **TPA** donor. (D) IPCE (Incident Photon-to-Electron Conversion Efficiency) results for FTO**|SnO2/TiO2|–RuIIP(TPA)(Cat)2+**|**AF**, at an applied bias of 0.6 V at pH 7.0 in a 0.1 M phosphate buffer. A 400 nm cut-off filter was used to mimic the conditions used in the current–time experiments.

The photo-stabilities of the assemblies, with the added 10–20 Å Dupont (**AF**) polymer overlayer, were evaluated by procedures described earlier.^82–84^ Derivatized electrodes were exposed to constant irradiation at 455 nm (fwhm ∼ 30 nm, 475 mW cm^−2^) in aqueous 0.1 M phosphonate at pH 7 solutions, 0.4 M in NaClO_4_. Absorption spectra (360–800 nm) were obtained every 15 min over 16 h periods of irradiation; results are shown in Fig. S9 and S10.† They demonstrate a significant enhancement in surface stability for the assemblies with the added aniline donor. As shown in Fig. 2A, following a 16 h irradiation period, the surface coverage of the chromophore FTO**|TiO2|–RuIIP2+|AF** had decreased to nearly zero but the decrease was only 50% for FTO**|TiO2|–RuIIP(TPA)2+|AF** (Fig. 2A).

Addition of the **TPA** donor stabilizes the excited state at pH 7. Earlier results on the transient FTO**|TiO2(e−)|–RuIIIP3+**, showed that it was unstable toward long term hydrolysis of the bipyridine ligands on Ru(iii) based chromophore.^77,85,86^ With the added triphenylamine derivative, excitation and quenching give FTO**|TiO2(e−)|–RuIIIP(TPA)3+|AF**. The latter is followed by transfer of the oxidative equivalent to the triphenylamine derivative to give **|–RuIIP2+(TPA+˙)3+|AF**, with the latter stabilizing the transient excited state.

### Water oxidation

Core–shell SnO_2_/TiO_2_ electrodes, with 1–2 nm overlayers of the external polymer film **AF**, as described above, were used as photoanodes in photoelectrochemical water splitting cells. The photocurrent response with and without the added **TPA** electron donor was comparable for FTO**|SnO2/TiO2|–RuIIP(Cat)2+**|**AF** and FTO**|SnO2/TiO2|–RuIIP(TPA)(Cat)2+**|**AF**. Water oxidation was investigated by using a standard three-electrode photoelectrochemical cell with 1 sun illumination (100 mW cm^−2^, 400 nm cut off filter) in 0.1 M phosphonate buffers in 0.4 M NaClO_4_ at pH 7.0. The working electrodes were integrated with a platinum wire counter electrode and a Ag/AgCl (3 M NaCl) reference electrode.

As shown by the data in Fig. 2B, a comparison of photocurrents for FTO**|SnO2/TiO2|–RuIIP(TPA)(Cat)2+**|**AF** and **|–RuIIP(Cat)2+**|**AF**, at early times, shows that initial photocurrents were higher for the donor-free electrode but that they decreased by a factor of ∼2 over a period of minutes. With the donor-containing photoelectrode, the photocurrent increased slightly over the initial stages in the water oxidation cycle and reached a maximum at 0.58 mA cm^−2^. From the data in Fig. 2C, comparison of long-term photocurrents with and without the added electron transfer donor, is notable. It points toward an important role for the added electron transfer donor to impart an element of stability to the assembly on the electrode surface.

The stabilities of the photoanodes and their ability to produce O_2_ for extended periods was explored by using a collector–generator, dual working electrode.^87–90^ For FTO**|SnO2/TiO2|–RuIIP(TPA)(Cat)2+**|**AF**, O_2_ appeared as a product with a 83% efficiency over an electrolysis period of 3 h, Fig. S11.† After 3 h of continuous illumination, the assembly had a photocurrent density of 0.12 ± 0.02 mA cm^−2^, Fig. 2. As a control, the electrode FTO**|SnO2/TiO2|–RuIIP(Cat)2+**|**AF** had a photocurrent density of 85 μA cm^−2^ and an O_2_ production efficiency of 75% for 1 hour measurements under the conditions described above. A slight photocurrent density increase was noted at the beginning of the test due to local ionic equilibration.

Incident photon-to-current efficiency (IPCE) measurements, as a function of excitation wavelength, for FTO**|SnO2/TiO2|–RuIIP(TPA)(Cat)2+**|**AF**, at an applied bias of 0.6 V *vs.* NHE, are shown in Fig. 2D. The IPCE profiles overlap with the MLCT absorption profile for the chromophore, consistent with solar conversion initiated by light absorption by the chromophore. Based on the data in Fig. 2D, the IPCE value for FTO**|SnO2/TiO2|–RuIIP(TPA)(Cat)2+**|**AF** was 10.9% at the absorption maximum for the assembly at 460 nm.

### Photo-physics

Transient absorption measurements were used to understand the events occurring after MLCT excitation of the assemblies: FTO**|SnO2/TiO2|–RuIIP2+|(AF)**, FTO**|SnO2/TiO2|–RuIIP(TPA)2+|(AF)**, and FTO**|SnO2/TiO2|–RuIIP(Cat)2+|(AF)**, and of the complete assembly, FTO**|SnO2/TiO2|–RuIIP(TPA)(Cat)2+|(AF)**. As noted below, in analyzing the data, the majority of microscopic events following **–RuIIP2+** excitation occur on the sub-microsecond timescale. Based on previous studies on **–RuIIP2+**, and of assemblies on **SnO2/TiO2** core–shell electrodes, at the ∼4–5 nm thickness of the outer **TiO2** shell used in the core–shell experiments, the excited electron is largely trapped in the initial **TiO2** layer on the sub-microsecond timescale.^74,91,92^

Transient excitation of FTO**|SnO2/TiO2|–RuIIP2+|(AF)** at 400 nm occurs with the instantaneous bleach of the metal-to-ligand charge transfer absorption for the Ru(ii) chromophore at 470 nm to give the excited state, –RuIIP2+*. On the inert oxide matrix ZrO_2_, free of surface quenching events, the lifetime of the excited state, was ∼55 ns, Fig. S12 and Table S1†. On FTO**|SnO2/TiO2|–RuIIP2+|(AF)**, the excited state undergoes rapid electron injection into the oxide film, followed by back electron transfer, FTO**|SnO2/TiO2(e−)|–RuIIIP2+|(AF)** → FTO**|SnO2/TiO2|–RuIIP2+|(AF)**. As found in earlier studies on related surfaces, the kinetics for both electron injection and back electron transfer were biphasic, see below.^70,93^

**Fig. 3:**
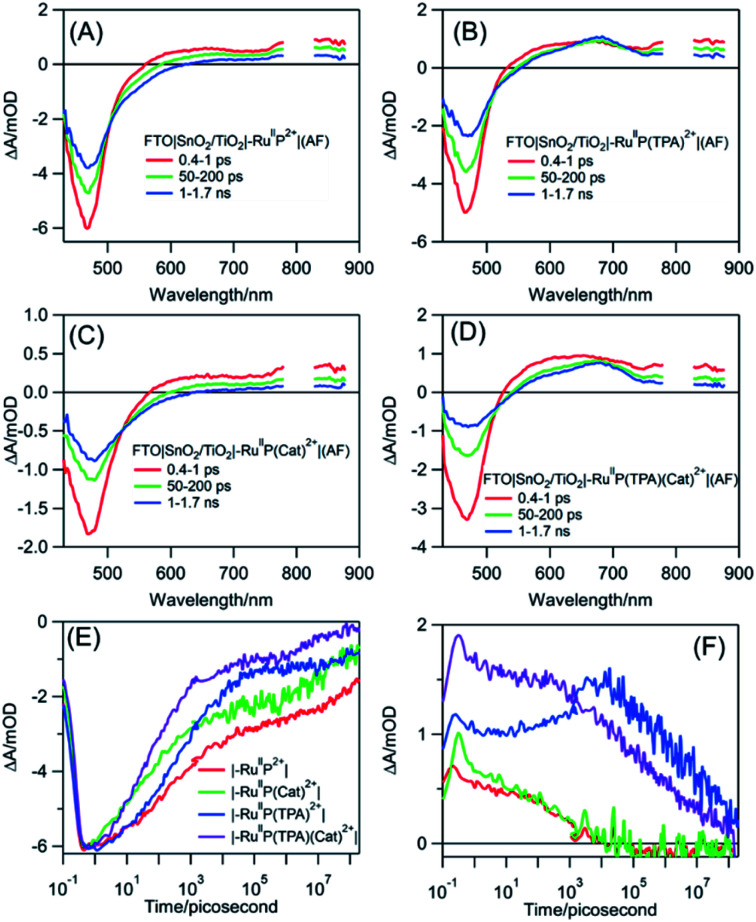
Transient absorption spectra for the assemblies, (A) FTO**|SnO2/TiO2|–RuIIP2+|(AF)**, (B) FTO**|SnO2/TiO2|–RuIIP(TPA)2+|(AF)**, (C) FTO**|SnO2/TiO2|–RuIIP(Cat)2+|(AF)**, and (D) FTO**|SnO2/TiO2|–RuIIP(TPA)(Cat)2+|(AF)**. Kinetics were evaluated at 470 nm at the ground state bleaches, note (E), and at 680 nm, in (F), for absorption by the **TPA** radical. The samples were excited at 400 nm with pulse energies of 100–300 μJ cm^−2^ in air.

Following excitation, **TiO2(e−)** has an intra-band absorption in the mid infrared. Observations in this spectral region were carried out by transient visible pump/mid-IR probe experiments on CaF_2_**|SnO2/TiO2|–RuIIP2+|(AF)**, Fig. S13.†^94–96^ Quantitative analysis of the kinetics data, probed at 5 μm, gave a time constant of 124 fs for electron injection and 56 ps for decay due to recombination and trapping. As noted in earlier transient studies in the visible, recombination can be monitored by following the recovery of the ground state bleach.^44,58^ Transient studies showed that 30% of the bleach recovery occurred with a 56 ps time constant, consistent with the IR decay data (Fig. S13C†). The remaining decay component that was observed arises from back electron transfer to the oxidized chromophore, **|–RuIIIP3+**, on the nanosecond to microsecond timescales, Fig. 3E and S14.† Analysis of the data, based on a KWW analysis, gave a lifetime of, *τ* = 188 ± 5 μs.

The donor-containing assembly, FTO**|SnO2/TiO2|–RuIIP(TPA)2+|AF**, was investigated to explore the kinetics of electron transfer from the oxidized sensitizer to **TPA**. Ultrafast excitation of FTO**|–RuIIP(TPA)2+|(AF)** results in electron transfer from the sensitizer to the electrode. Excitation is followed by intra-assembly electron transfer to the **TPA** donor to give FTO**|SnO2/TiO2(e−)|–RuIIP(TPA+˙)3+|(AF)**. The appearance of **TPA+˙** as an intermediate was shown by the appearance of a transient absorption feature with a maximum at ∼680 nm, Fig. 3B.^57^ The sequence of events following excitation is summarized in eqn (7)–(9). As noted below, the final back electron transfer in eqn (9) is sufficiently slow, that it follows after internal electron transfer equilibration in the core/shell.

7 FTO|SnO2/TiO2|–RuIIP(TPA)2+*|(AF) → FTO**|SnO2/TiO2(e−)|–RuIIIP(TPA)3+|(AF)**

8 FTO**|SnO2/TiO2(e−)|–RuIIIP(TPA)3+|(AF)** → FTO**|SnO2/TiO2(e−)|–RuIIP(TPA+˙)3+|(AF)**

9 FTO**|SnO2/TiO2(e−)|–RuIIP(TPA+˙)3+|(AF)** → FTO**|SnO2(e−)/TiO2)|–RuIIP(TPA+˙)3+|(AF)** → FTO**|SnO2/TiO2|–RuIIP(TPA)2+|(AF)**

In comparing transient results, the appearance of the ground state bleach in FTO**|SnO2/TiO2|–RuIIP(TPA)2+|AF**, Fig. 3E, is decreased in magnitude compared to FTO**|SnO2/TiO2|–RuIIP2+|(AF)**. The decrease is due to hole transfer in the aniline-containing assembly from the oxidized chromophore to the aniline donor. Based on an analysis of the data in Fig. S15,† oxidation of the initial transient, FTO|**SnO2/TiO2(e−)|–RuIIIP(TPA)3+** to FTO|**SnO2/TiO2(e−)|–RuIIP(TPA+˙)3+**, eqn (8), occurs with a lifetime of ∼830 ps. Back electron transfer from the electrode to the external aniline cation to give the ground state, eqn (9), occurs following internal electron equilibration of the core–shell with a lifetime of ∼17 μs, Fig. S15C.†

Given the spectral properties of the catalyst, in the catalyst-containing assembly, **FTO|SnO2/TiO2|–RuIIP(Cat)2+|(AF)**, there were no spectral probes for the direct observation of hole transfer from the excited state to the catalyst, FTO**|–RuIIIP(Cat)3+|(AF)** → FTO**|–RuIIP(Cat′)3+|(AF)**. However, the ground state bleach recoveries in Fig. 3B, C, E and S16† are more rapid than in **FTO|SnO2/TiO2(e−)|–RuIIIP|3+(AF)**. The latter is consistent with hole transfer to the catalyst and the reaction sequence in eqn (10)–(12). Analysis of the kinetics in Fig. S16B,† assuming return of the added electron from hole transfer to the catalyst, gave a lifetime of ∼28 ps. The lifetime for back electron transfer to the oxidized catalyst, following internal equilibration of the core/shell, eqn (12), was ∼64 μs, Fig. S16A.†

10 FTO|SnO2/TiO2|–RuIIP*(Cat)2+|(AF) → FTO**|SnO2/TiO2(e−)|–RuIIIP(Cat)3+|(AF)**

11 FTO**|SnO2/TiO2(e−)|–RuIIIP(Cat)3+|(AF)** → FTO**|SnO2/TiO2(e−)|–RuIIP(Cat′)3+|(AF)**

12 FTO**|SnO2/TiO2(e−)|–RuIIP(Cat′)3+|(AF)** → FTO**|SnO2/TiO2|–RuIIP(Cat)2+|(AF)**

In the complete assembly, FTO**|SnO2/TiO2|–RuIIP(TPA)(Cat)2+|(AF)**, transient data were used to investigate hole transfer between **TPA+˙** and the catalyst, eqn (14), by the excitation–reaction sequence in eqn (13)–(15). In evaluating the dynamics for intra-assembly electron transfer, FTO**|SnO2/TiO2(e−)|–RuIIIP(TPA)(Cat)3+|** → FTO**|SnO2/TiO2(e−)|–RuIIP(TPA+˙)(Cat)3+|**, eqn (14), decay of the **TPA+˙** at 680 nm, Fig. S17† and 3F, occurs by a more rapid, ground state bleach recovery, Fig. 3E. Based on the data, an estimate for the timescale for hole transfer from the catalyst to TPA of ∼3.6 ns was obtained by analysis of the TPA radical kinetics. The data are shown in Fig. S17.†

13 FTO**|SnO2/TiO2(e−)|–RuIIIP(TPA)(Cat)3+|(AF)** → FTO**|SnO2/TiO2(e−)|–RuIIP(TPA+˙)(Cat)3+|(AF)**

14 FTO**|SnO2/TiO2(e−)|–RuIIP(TPA+˙)(Cat)3+|(AF)** → FTO**|SnO2/TiO2(e−)|–RuIIP(TPA)(Cat′)3+|(AF)**

15 FTO**|SnO2/TiO2(e−)|–RuIIP(TPA)(Cat′)3+|(AF)** → FTO**|SnO2/TiO2|–RuIIP(TPA)(Cat)2+|(AF)**

**Scheme 2:**
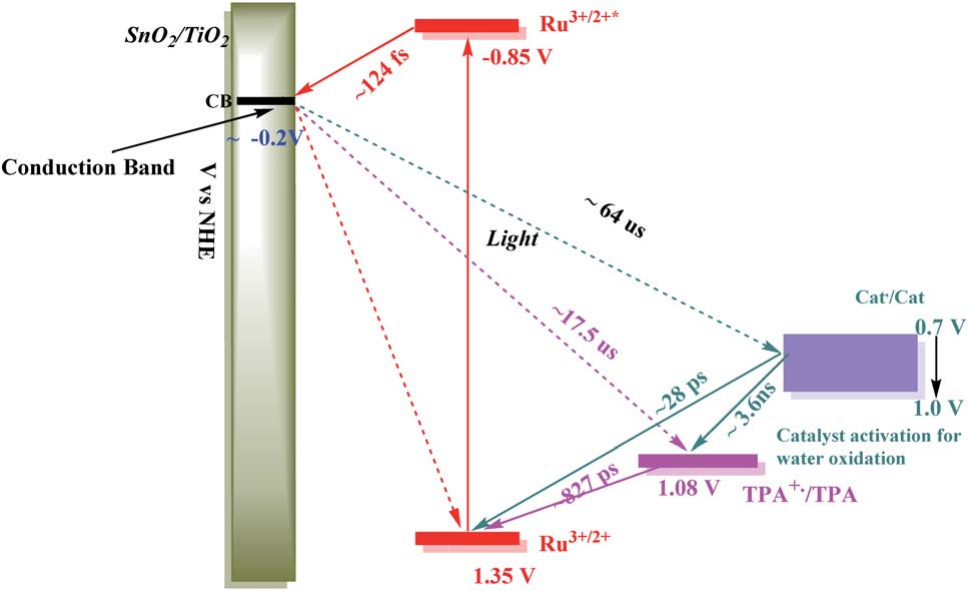
Redox potential diagram based on kinetic studies of the component assemblies and of the final assembly, FTO**|SnO2/TiO2|–RuIIP(TPA)(Cat)2+|(AF)***vs.* NHE, for the first step in the water oxidation cycle. The range of potentials for the three electrons transfer activation of the catalyst is also shown.

The kinetics data summarized here were limited to the first stage in the overall cycle for water oxidation assuming the reactions in eqn (13)–(15). Timescales and rate constants for the individual steps, as observed for the individual assemblies by TA measurements, are summarized in Scheme 2 and Table S3.† The exact role of the mediator, which may play a larger role in the 2^nd^, 3^rd^ or 4^th^ photoactivation steps of the catalyst, are difficult to ascertain from the current experiments. The initial one-electron oxidation of the catalyst from Ru(ii) to Ru(iii) is likely not a significant contributor to the device performance and can be readily achieved by the chromophore alone in the 1^st^ photoactivation step based on the current kinetic measurements. The TPA mediator likely plays a greater role in either activating the catalyst or storing oxidative equivalents in later stages of the water oxidation catalytic cycle not probed here.

## Discussion

A DSPEC was prepared and characterized with an integrated semiconductor–molecular assembly that mimicked PSII's ability to use visible light to drive water oxidation to O_2_. The core of the assembly utilized a derivatized polypyridyl complex of Ru(ii) which served as both the light absorber and as a scaffold for the assembly of multifunctional units for water oxidation.

Flash photolysis experiments on component assemblies, and on the final assembly, **|SnO2/TiO2|–RuIIP(TPA)(Cat)2+**, gave significant insights into the microscopic details that occur following MLCT excitation of the **–RuIIP2+** chromophore. Excitation of the assembly was the first step in its overall activation toward water oxidation. The results of flash photolysis experiments revealed a high level of electron transfer reversibility following the initial 1e^−^ oxidation of the **–RuIIP2+** chromophore with no evidence for decomposition of the assembly after repeated transient cycles. Decomposition of the assembly, over extended cycles, occurs following 3-electron oxidation of the catalyst with decomposition occurring in competition with the evolution of O_2_.^97^

An energy level diagram for the first step in the water oxidation cycle by the final assembly is shown in Scheme 2. The diagram includes estimates for the individual kinetic steps based on the results of lifetime measurements on the model complexes as discussed above. It also shows the range of redox potentials required for activation of the assembly to its activated 3e^−^ form. Based on the scheme, excitation of the **–RuIIP2+** chromophore is followed by excited state injection into the SnO_2_/TiO_2_ core/shell electrode with the core/shell inhibiting back electron transfer to the oxidized chromophore on the surface.^98^ Following excitation and injection, electron transfer occurs through the core/shell electrode to the cathode where H_2_ is formed.

Following the initial excitation step, injection and formation of **|–RuIIIP(TPA)(Cat)3+|AF** occurs within the assembly, followed by oxidation of the triphenylamine, gives, **|–RuIIP(TPA+˙)(Cat)3+|(AF)**. In the first stage of the water oxidation cycle, the latter undergoes internal electron transfer and loss of a proton to give the singly oxidized, **RuIII–OH2+** form of the catalyst, **Cat′**, in **|–RuIIP(TPA)(Cat′)2+|(AF)**. The latter is the first intermediate in the overall oxidation of water to O_2_.

As noted in the Introduction, tyrosine, and its accompanying base, play important roles in PSII as redox mediators between the chromophore and catalyst. A similar redox mediator role may also be played by the triphenylamine cation in **|–RuIIP(TPA+˙)(Cat)3+|(AF)**. Based on the known mechanistic chemistry of the catalyst in water oxidation in solution, we assumed the catalyst experienced a parallel process on surface. Once oxidized, it undergoes further oxidation through two additional cycles to reach Ru(v).^63,79^ Based on previous literature results, in the overall cycle, oxidation to Ru(v) is followed by coordination expansion and O⋯O bond formation through a transient peroxide intermediate. The latter undergoes further oxidation and loss of O_2_.^33,59,99^

For the catalyst in the assembly, 2e^−^ oxidation and proton loss give the intermediate, **|–RuIIP(TPA)(Cat′′)2+|(AF)**, with the catalyst oxidized to Ru^IV^(O)^2+^. An additional oxidative equivalent is required to give the active form of the catalyst. In the three-electron oxidized form of the assembly, **|–RuIIP(TPA+˙)(Cat′′)3+|(AF)**, the driving force required to reach the activated three electron oxidized form of the catalyst by TPA radical cation is only ∼0.1 V. Once reached, the active form proceeds through the series of steps required for O⋯O bond formation and water oxidation.

In the overall reaction sequence, the triphenylamine donor redox couple is a kinetically accessible kinetic intermediate. It, and the role that it plays, also provides insight into the role of the tyrosine–histidine acid–base pair in PSII. Intervention of the latter would explain the value of a separate step in which the redox equivalent for the final activation step is stored in the tyrosine–histidine acid–base pair with **|–RuIIP(TPA+˙)(Cat′′)2+|(AF)** as an analog. In the final step in the activation of the catalyst, as for tyrosine in PSII, the assembly is converted into an active form that provides access to a reactive form of the catalyst.

## Conclusions

The results described here describe a procedure for the preparation of a surface assembly that mimics the ability of PSII for using visible light for water oxidation to O_2_ with an electron transfer mediator. Although relatively simple compared to PSII, the assembly includes all of the key functional elements of PSII, including light absorption, electron transfer activation, catalysis, mediation, and the light-induced formation of O_2_. Notable in the results, as revealed by flash photolysis measurements, was the extensive series of electron transfer steps that occur in the light absorption cycle by the molecular chromophore and the creation and storage of redox equivalents in an attached catalyst for water oxidation.

## Data availability

All relevant data have been included in the ESI.†

## Author contributions

D. Wang and T. Meyer designed the study. D. Wang, Z. Xu performed the experiments. J. Concepcion and F. Li synthesized the molecular. D. Wang, Z. Xu and M. Sheridan analyzed the data. All authors made contribution to the discussions during the work. D. Wang, Z. Xu, M. Sheridan, T. Lian and T. Meyer prepared the manuscript. T. Meyer supervised the project.

## Conflicts of interest

There are no conflicts to declare.

## Supplementary Material

SC-012-D1SC03896F-s001
