# Supplementary material for: Photodriven water oxidation initiated by a surface bound chromophore-donor-catalyst assembly
Source: Chem Sci. 2021 Oct 11;12(43):14441–50. doi: 10.1039/d1sc03896f (PMC8580115; doi:10.1039/d1sc03896f)
Supplement: SC-012-D1SC03896F-s001 [file SC-012-D1SC03896F-s001.pdf]

Electronic Supplementary Material (ESI) for Chemical Science.
This journal is © The Royal Society of Chemistry 2021

Supporting Information

Materials.

All chemicals are used as received without further purification.

The chromophore-catalyst assembly was synthesized as reported.[1] And the electron transfer mediator TPA was synthesized as described in the literature.[2-3]

Preparation of SnO_2 Films. SnO_2 paste was prepared by using colloidal suspension (15% w/v) of SnO_2 particles from Alfa Aesar. In the procedure, 1 g of acetic acid was added to 37 g of a SnO_2 colloidal solution to prepare the paste. The resulting mixture was sealed and stirred overnight. In the next step, the as-prepared solution was transferred to an autoclave (100 mL volume). The autoclave was sealed and placed in a box oven and heated for 45 min at 240° for 80 h. After the mixture was cooled, the colloids were redispersed in a sonic hall from Branson Ultrasonics for 2 min. To the dispersed mixture was added mixed polyethylene oxide (mol wt 100,000) and polyethylene glycol (mol wt 12,000). The resulting mixture was covered and stirred a further 48 h. A doctor-blading method was utilized to make the SnO_2 film. The slides were annealed at 450° for 1 h to give 4~5-μm, 20-nm SnO_2 nanoparticle films.

Preparation of TiO_2 , nanoITO and ZrO_2 mesoporous films are according to the reported procedure.[4-5]

Atomic Layer Deposition. ALD was performed by using a Cambridge NanoTech Savannah S200 instrument with TDMAT [tetrakis(dimethylamino)titanium] as Ti precursor. ALD was performed in a commercial reactor (Savannah S200; Cambridge Nanotech). The chamber pressure is 760 torr when exposed to air. Titanium dioxide (TiO_2) was deposited with Tetrakis (dimethylamido) titanium, Ti $(NMe_2)_4$ (TDMAT, 99.999%; Sigma-Aldrich), and water. The reactor chamber temperature was 150 °C. The TDMAT reservoir was kept at 75 °C (2-h preheating). ALD coating conditions were 150 °C and 20 torr of N_2 carrier gas with a sequence of 0.5-s metal precursor dose, 20-s hold, 30-s N_2 purge, 0.02-s H2O dose, 20-s hold, 30-s N_2 purge. After deposition of 75 cycles of TiO_2 on SnO_2 films, the slides were placed into box oven for 30 min with annealing under 450°.

Preparation of Electrodes. The FTO|SnO_2/TiO_2 slides were prepared by immersing the slides into molecular solutions (4 mM) with soaking for 12 h. The molecular-loaded slides were 5mM $Zr(OCl)_2$ methanol solution for 2 hours to link Zr^{IV}. After depositing Zr^{IV}, the slides were immersed in 2 mM CH_2Cl_2 solutions of TPA for an additional 1 h followed by rinsing with CH_2Cl_2.The as prepared slides was stored in Glovebox for further use. For other slides preparation, using different substrate but similar procedure.

UV-Visible spectroscopy.

Agilent Technologies Cary 8454 UV-visible spectrometer was used to perform UV-visible absorbance measurements.

Photoelectrochemical Measurements. Electrochemical and photoelectrochemical experiments were performed by using either a CH Instruments 660D potentiostat or a CH Instruments 760E bipotentiostat. A Thor Labs HPLS 30–04light source was used to provide white-light illumination. For all indicated experiments using 100-mW cm^{-2} white-light illumination, the electrochemical cell was positioned an appropriate distance from the light source to receive the indicated light intensity as measured with a photodiode (Newport), and a 400-nm cutoff filter (Newport) was used to prevent direct bandgap excitation of the semiconductor layer. In the water oxidation experiments, a two-compartment cell with a Nafion membrane was used in a three-electrode configuration, with Ag/AgCl as the reference electrode and a Pt mesh counter electrode for H_2 evolution. The experiments were carried out under N_2 at pH = 7.0 in a 0.1 M aqueous sodium phosphate buffers in 0.4 M $NaClO_4$ with a 100-mW/cm^2 white-light source (400-nm cutoff filter) at a bias of 0.4 V versus Ag/AgCl. For the electrode, we have used 3 samples to test FTO|SnO_2/TiO_2|**-Ru^{II}P (TPA)(Cat)$^{2+}$** water oxidation stability.

IPCE Measurement.

It is well-known that the potential of the Ag/AgCl reference (saturated KCl) is constant with respect to absolute potentials such as NHE (+0.1976 V at 298 K). Incident photon-to-current conversion efficiencies (IPCEs) were measured in a similar manner using a 300 W xenon lamp with a band-pass filter. In all cases, we confirmed that the Ag/AgCl reference electrode correctly worked during the photoelectrochemical experiments, by measuring the potential difference

between the used Ag/ AgCl reference electrode and another fresh one. For the electrode, we have used 3 samples to test **FTO|SnO_2/TiO_2|-Ru^{II}P (TPA)(Cat)$^{2+}$** IPCE value.

Quantifying O_2 Evolution. To quantify the amount of evolved O_2, generation, collector–generator (C-G) experiments were carried out with a dual-electrode design described elsewhere (31, 32). The results of a 1-h illumination period with an ∼1-sun intensity light source (100-mW cm−2 and 400-nm long-pass filter). At the end of a photolysis cycle, the generator current decayed instantaneously with slower decay at generator electrode as diffusion of O_2 to the electrode occurs. Faradaic efficiencies (FE) for O_2 production were calculated with Qcollector and Qgenerator the total charge passed at the collector and generator electrodes, respectively. The constant, 0.7, is the experimentally derived, collection efficiency for the cell, as described previously.

XPS spectra were acquired by using a Kratos Axis Ultra DLD X-ray photoelectron spectrometer with a base pressure of 6 × 10−9 Torr, a monochromatic Al Kα X-ray source, and an analyzer pass energy of either 80 eV or 20 eV for survey and high resolution scans respectively.

Photostability Measurement.

Derivatized electrodes were exposed to constant irradiation at 455 nm (fwhm ∼30 nm, 475 mW/cm^2, ∼135 suns at 455 nm) in aqueous 0.1 M phosphonate at pH 7 solutions, 0.4 M in $NaClO_4$. Absorption spectra (360−800 nm) were obtained every 15 min over 16 h periods of irradiation.

(Transmission Electron Microscopy) TEM analysis was performed on a JEOL 2010F FasTEM by applying an accelerating voltage of 200 kV.

A FEI Helios 600 Nanolab Dual Beam System focused ion beam (FIB) was used to acquire **Scanning electron microscope (SEM)** images.

Transient absorption spectroscopy.

The femtosecond to microsecond visible pump, visible probe transient absorption spectroscopy is based on a 1 kHz Ti:Sapphire 150 fs ultrafast laser (Coherent, Legend). Part of the fundamental 800 nm was doubled by a BBO crystal to generate the 400 nm excitation light, part of the fundamental 800 nm was focused on a sapphire plate to generate whitelight as probe in the femtosecond to nanosecond time window. Beyond this time window, the probe light is generated by a 2 kHz supercontinuum laser. The pump and probe are focused onto the sample, where they

coincide both in time and space. The probe light after the sample is collected by focal lens and optical fiber, before they are diffracted by a grating and detected by a CMOS array. The controlling software is Helios and EOS from Ultrafast Systems.

The femtosecond visible pump, mid infrared transient absorption spectroscopy is based on a 1 kHz Ti:Sapphire 35 fs ultrafast laser (Coherent, Astrella). The pump is generated by a visible OPA (Coherent, OPerA), the probe is generated by a mid-IR OPA (Coherent, OPerA). The geometry shares similarity with visible setup. The probe light is collected by parabolic mirror and diffracted by a grating, before detected by a liquid nitrogen cooled MCT detector.

Supplementary results

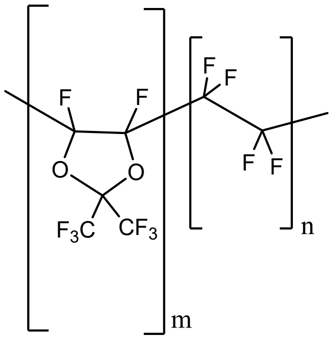

Figure S1. The structure of polymer, 4,5-difluoro-2,2-bis(trifluoromethyl)-1,3-dioxole **(AF)**

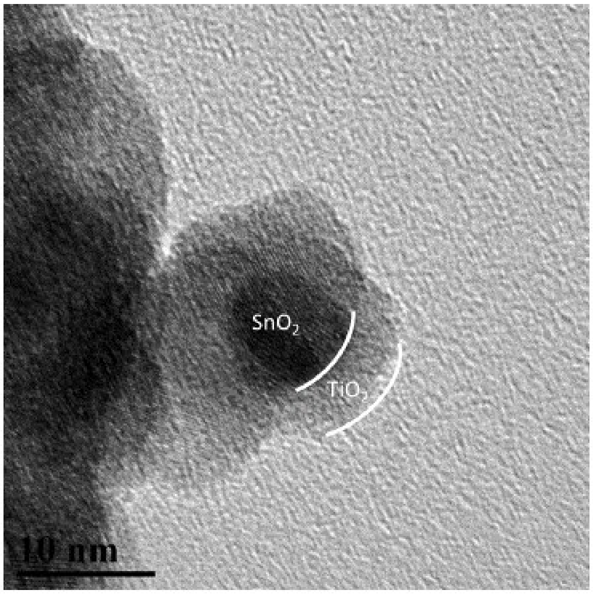

Figure S2. Transmission electron microscope image of SnO_2 coated by a 4.5-nm TiO_2 shell deposited by ALD.

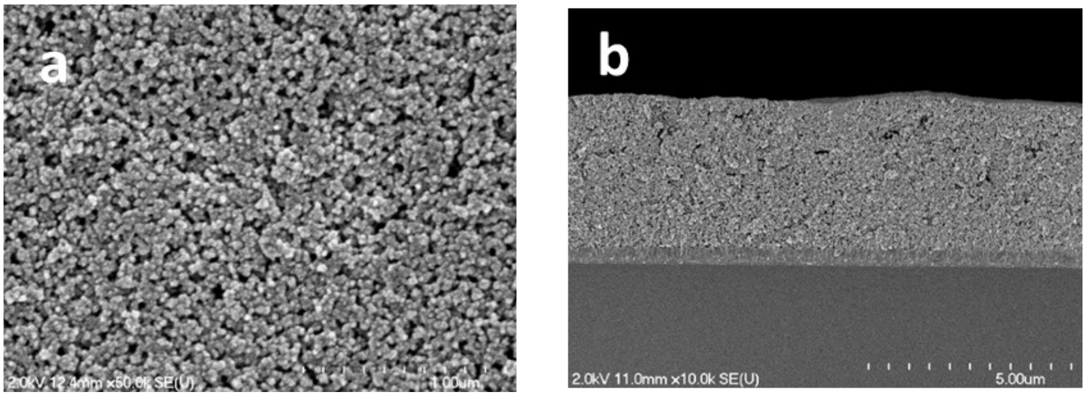

Figure S3. SEM images of the photoanode surface. (a) Top view of the FTO|SnO_2/TiO_2 film. (b) Cross-sectional view of the same electrode.

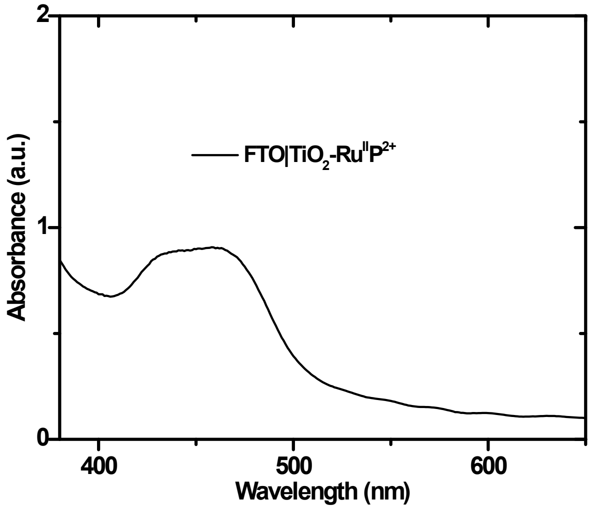

Figure S4. Absorption spectra for FTO|TiO$_2$|-**Ru^{II}P^{2+}**, obtained at room temperature in air.

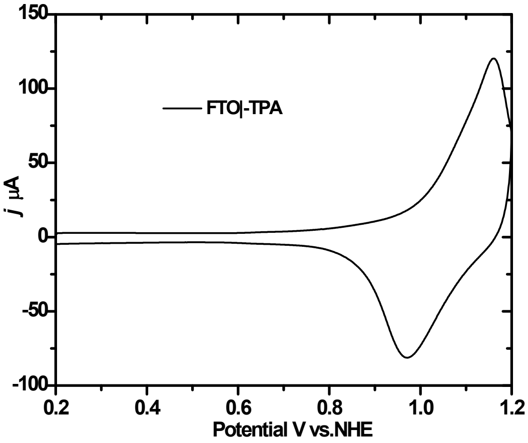

Figure S5. Cyclic voltammograms for **TPA** on planar FTO in pH 7.0 in 0.1M phosphonate buffers, in 0.4 M in NaClO$_4$ at 50 mV/s.

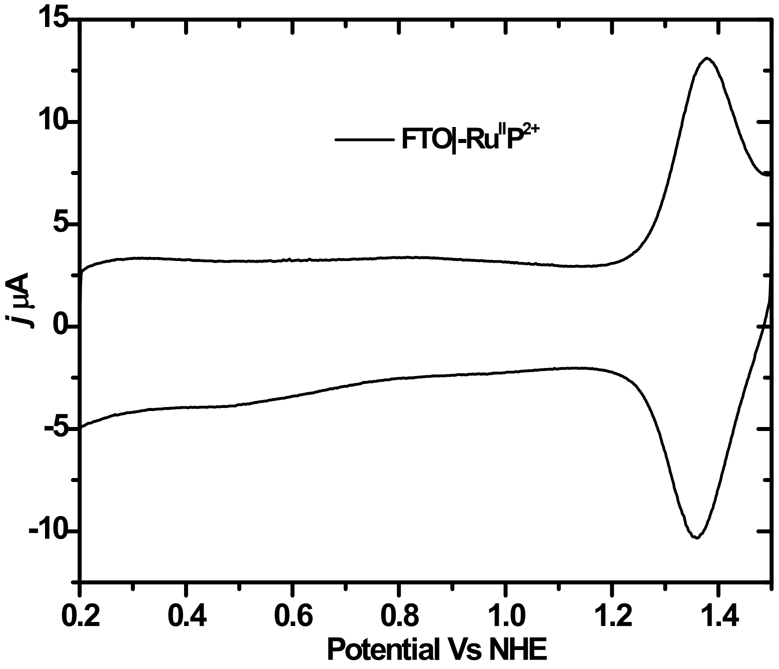

Figure S6. Cyclic voltammograms for **$Ru^{II}P^{2+}$** on planar FTO in pH 7.0 in 0.1M phosphonate buffers, in 0.4 M in $NaClO_4$ at 50 mV/s.

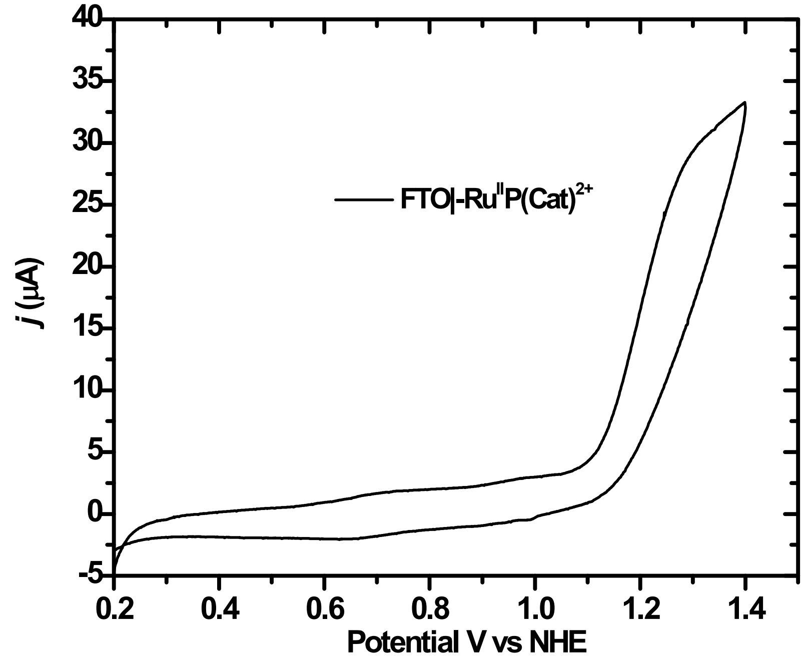

Figure S7. Cyclic voltammograms for 1 **Ru^{II}P(Cat)** $^{2+}$on planar FTO in pH 7.0 in 0.1M phosphonate buffers, in 0.4 M in $NaClO_4$ at 50 mV/s.

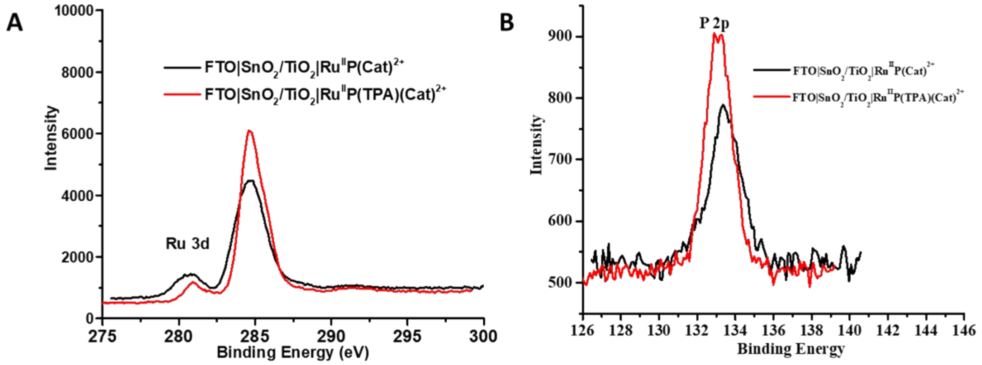

Atom Peak	Ru 3d	P 2p	Ratio Ru/P
**FTO|SnO_2/TiO_2| Ru^{II}P(Cat)$^{2+}$**	0.74	1.35	0.55
**FTO|SnO_2/TiO_2|Ru^{II}P(TPA)(Cat)$^{2+}$**	0.73	2.25	0.33

Figure S8. Quantification of XPS signals for the indicated assemblies used to calculate the chromophore-catalyst/TPA ratio. The ratio of chromophore-catalyst/TPA in the **FTO|SnO_2/TiO_2| Ru^{II}P(TPA)(Cat)$^{2+}$** assemblies is ~1:1 using the change in the ratio of Ru/Pbetween samples with and without added TPA.

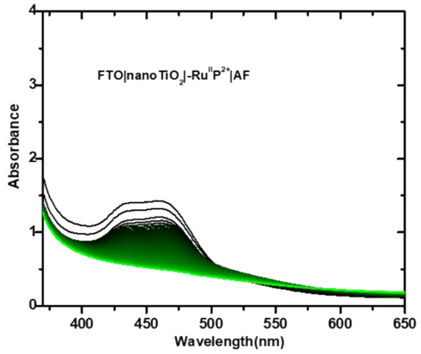

Figure S9. Changes in the absorption spectrum of **FTO|nanoTiO$_2$|-Ru^{II}P^{2+}|AF** in aqueous pH 7.0 with 0.4 M NaClO$_4$ under constant 455 nm irradiation (475 mW/cm^2) from 0 (black) to 16 h (green) recorded every 15 min.

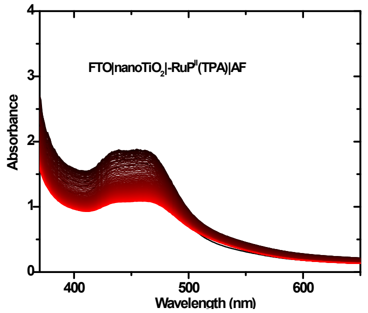

Figure S10. Changes in the absorption spectrum of **FTO|nanoTiO$_2$|-Ru^{II}P (TPA)$^{2+}$|AF** in aqueous pH 7.0 with 0.4 M NaClO$_4$ under constant 455 nm irradiation (475 mW/cm^2) from 0 (black) to 16 h (red) recorded every 15 min.

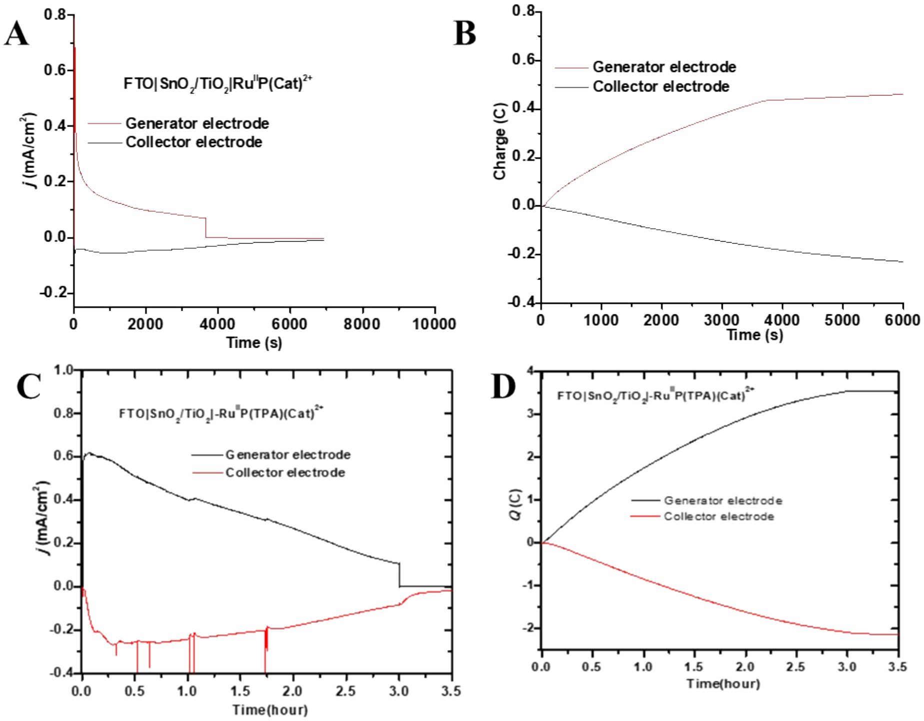

Figure S11. O_2 measurements for water oxidation from (A, B) FTO|SnO_2/TiO_2**|-Ru^{II}P (Cat)$^{2+}$** (C, D) FTO|SnO_2/TiO_2**|-Ru^{II}P (TPA)(Cat)$^{2+}$** on 1-cm^2 slide illuminated with a 100 mW·cm^{-2} white light with a 400-nm cutoff filter over aperiod at a bias of 0.6 V vs. NHE and an FTO electrode positioned 1 mm from the photoanode poised at −0.65 V vs. NEH. The experiment was performed in 0.1 M acetic acid/acetate buffer at pH 7.0 with 0.4 M $NaClO_4$. The integrated current over time (charge) used to calculate Faradaic efficiencies for the same collector and generator electrodes shown in Left.

In the following procedures to determine the time constant for the electron transfer processes, some of them are not fitted with a single exponential function. Alternatively, the kinetics of the resulting transients were satisfactorily modeled by application of the Kohlrausch-William-Watts (KWW) function. The value of β in the analysis was inversely related to the breadth of the corresponding Lévy distribution function. Rate constants, k_{cr}, were calculated from the expression, $A(t)=\Delta A_0\exp(-(kt)^{\beta})$, in which A(t) is the transient absorbance change, ΔA the total absorbance change, k the experimental rate constant, and β the exponential rate term. The origin of the stretched exponential kinetics that were observed may result from dispersive transport of charge carriers with the transport rate-limited by thermally activated, trapping and de-trapping events.[6] In the following measurement and fitting, the lifetime (time constant τ) and rate (k) are used, their relationship is $k=1/\tau$.

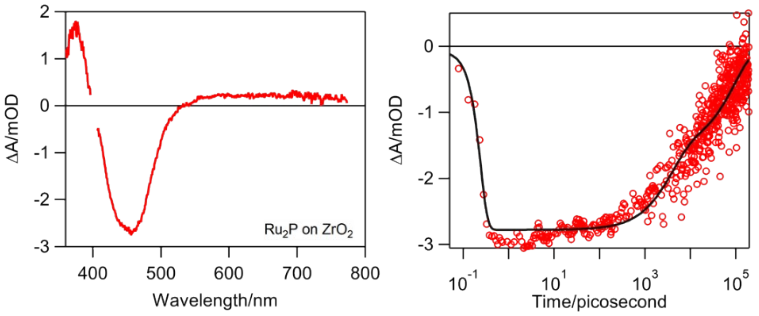

Figure S12. Left: **$Ru^{II}P^{2+}$** visible transient spectrum averaged from 1-1000 ps after 400 nm excitation. Right: 470 nm kinetics of **$Ru^{II}P^{2+}$** on ZrO_2 after 400 nm excitation. The excited state lifetime of **$Ru^{II}P^{2+}$** could be determined by visible pump probe transient spectroscopy on a ZrO_2 substrate, where neither electron nor hole transfer is allowed energetically. The lifetime of **$Ru^{II}P^{2+}$** excited lifetime could be fitted by a two-exponential function. The τ_4 could be determined to be 54.9±6.5 ns with the fitting parameters shown in **Table S1**.

Table S1. Fitting result for **$Ru^{II}P^{2+}$** on ZrO_2 in **Figure S12**.

irf_FWHM	0.215 ± 0.043 ps
t0	-0.207 ± 0.013 ps
a0	-1.24 ± 0.05 mOD
tau0	3.80 ± 0.45 ns

a1	-1.54 ± 0.05 mOD
tau1	96.0 ± 5.4 ns

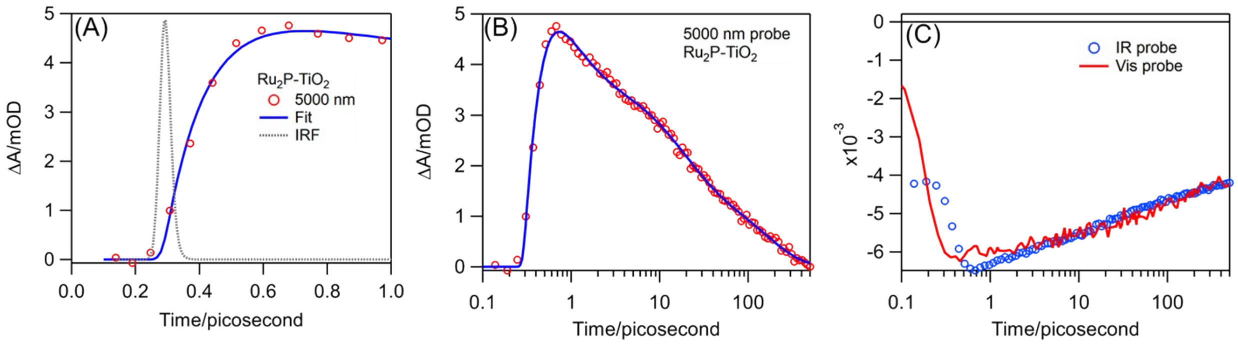

Figure S13. Visible pump at 400 nm, mid-IR probe kinetics at 5000 nm on CaF_2|**SnO_2/TiO_2|-$Ru^{II}P^{2+}$** thin film. (A) The grey line shows the instrument response function. (B) The decay is fitted to a multi exponential decay with the rate constant shown in **Table S2**. (C) The IR probe kinetics agrees with the fast decay of the visible probed GSB kinetics. The determination of k_1 and k_5 by visible pump, IR probe transient for electron injection into TiO_2 is described below.

In order to determine the electron injection rate k_1 and recombination rate k_4, a **$Ru^{II}P^{2+}$** sample was prepared on CaF_2 substrate and excited by 400 nm excitation light. The probe was selected to be at 5000 nm to probe the electron intraband transition in TiO_2's conduction band. As shown in **Figure S13**, the electron injection in TiO_2 results in the growth of signal at a sub picosecond time

scale. This signal decays at tens of picosecond. The growth and decay of this signal could be fitted with one exponential function for growth and three exponential functions for decay convoluted with instrument response function. The fitting results are shown table below. Therefore, τ_1=124±13 fs and τ_5=56.0±9.1 ps. The recombination rate by **Ru^{II}P** τ_4 is determined by replacing the electron accepting TiO_2 with insulating ZrO_2. The associated time constant is shown in **Figure S12** and the **Table S1** below. It is worth noticing that the recombinateion discussed here is biphasic, with the fast recombination rate constant determined here. The slower recombination rate constant is determined in **Figure S12**. The origin of biphasic recombination is attributed to different location of electron in TiO_2/SnO_2.

Table S2. Fitting result for CaF_2|**SnO2/TiO_2**|**Ru^{II}P^{2+}** in **Figure S13**.

irf_FWHM	0.035 ps
t0	-0.284 ps
a0	-5.47 ± 0.05 mOD
tau0	0.124 ± 0.013 ps
a1	1.66 ± 0.01 mOD
tau1	0.92 ± 0.15 ps
a2	2.08 ± 0.15 mOD
tau2	16.7 ± 1.4 ps
a3	1.75 ± 0.08 mOD
tau3	154.9 ± 9.3 ps

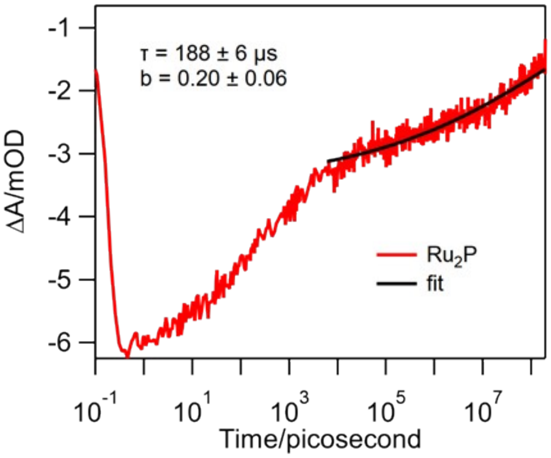

Figure S14. Charge separation state of **-Ru^{III}P^{3+}**recombination time constant, τ_5, determined by KWW function to be 188±5 μs.

The GSB kinetics probed at 470 nm of the **-Ru^{II}P^{2+}** sample provides a recombination of the charge separation state (FTO|SnO$_2$/TiO$_2$(**e**$^-$)|**-Ru^{III}P^{3+}**), which can be fitted by KWW function as shown in **Figure S14**. The time constant corresponds to the slower phase recombination of electron in TiO$_2$ and hole in **Ru^{II}P^{2+}**.

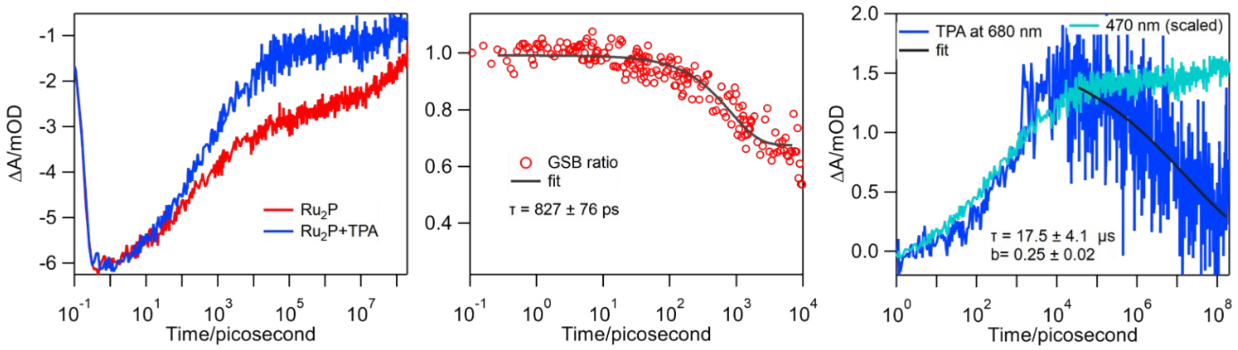

Figure S15. Determination of hole transfer rate to **TPA** shown in the left panel, the fitting of τ_2 is shown in the middle panel and the fitting of τ_6 is shown in the right panel. In the right panel, the contribution from Ru(II) is subtracted by normalizing the signal contribution at 680 nm of Ru(II) to the GSB signal at 470 nm.

Comparing the GSB kinetics of **-Ru^{II}P^{2+}** and **-Ru^{II}P(TPA)$^{2+}$** on FTO|SnO_2/TiO_2, the hole transfer with the rate constant τ_2 could be determined. The GSB of Ru^{II}P^{2+} could be described by $e^{-(k4+k5)t}$ where **-Ru^{II}P(TPA)$^{2+}$**could be described by $e^{-(k2+k4+k5)t}$. In order to simplify the fitting procedure, we divide these two GSBs and fit the result with a single exponential function to obtain τ_2. Subsequently, the **-Ru^{II}P(TPA$^{+\cdot}$)$^{3+}$**lifetime τ_6 could be determined as well by fitting the decay of the TPA$^{+\cdot}$ signal at 680 nm as shown in **Figure S15** with KWW function as shown in the right panel. The obtained TPA radical kinetics growth agrees well with the initial decay of the GSB of **-Ru^{II}P(TPA)$^{2+}$** after scaling as shown in the right panel. This proves the TPA radical is a product of the hole transfer from **Ru^{II}P^{2+}**.

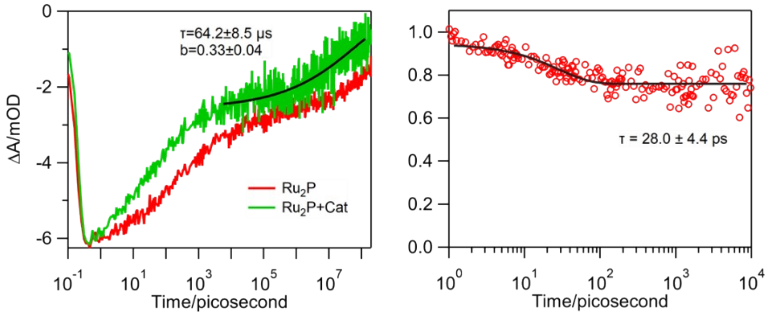

Figure S16. Determination of τ_8. Comparison of GSB kinetics of **-Ru^{II}P^{2+}** and **-Ru^{II}P(Cat)$^{2+}$** could determine the hole transfer to catalyst rate τ_8.

Similarly, comparing the GSB kinetics of **-Ru^{II}P^{2+}** and **-Ru^{II}P(Cat)$^{2+}$** on FTO|SnO_2/TiO_2, the hole transfer with the rate constant k_8 could be determined. The GSB of **Ru^{II}P^{2+}** could be described by $e^{-(k4+k5)t}$ where **-Ru^{II}P(TPA)$^{2+}$**could be described by $e^{-(k8+k4+k5)t}$. In order to simplify the fitting procedure, similar to the treatment in **Figure S15**, we divide these two GSBs and fit the result with a single exponential function to obtain τ_8. The recombination rate for hole in catalyst τ_7 is also fitted by a KWW function and the result is 64.2±8.5 μs.

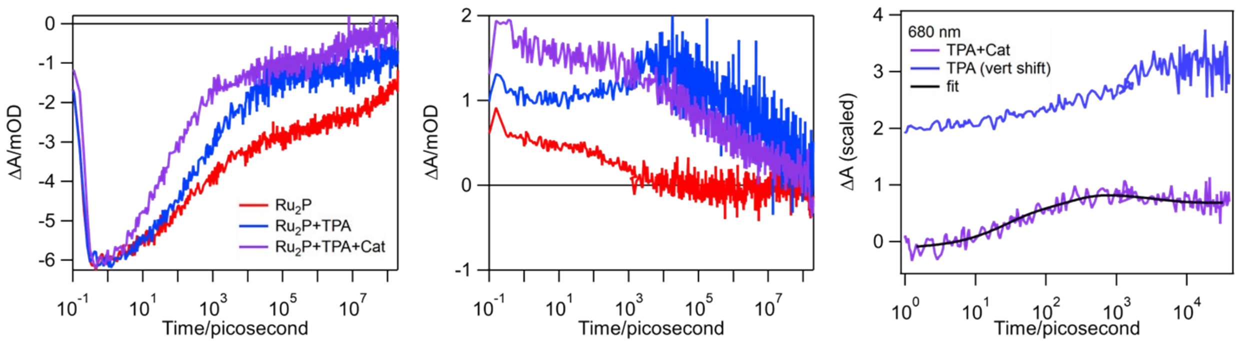

Figure S17. Left panel: kinetics at 470 nm for **-Ru^{II}P^{2+}** (red), **-Ru^{II}P(TPA)$^{2+}$** (blue) and -**Ru^{II}P(TPA)(Cat)$^{2+}$** (purple). Middle panel: kinetics at 680 nm of the same samples with same color legends. Right panel: kinetics at 680 nm after removal of **-Ru^{II}P^{2+}** contribution, the kinetics in the right panel represents the pure **TPA$^{+\cdot}$** kinetics.

The **TPA$^{+\cdot}$** signal at 680 nm is used to determine the hole transfer from **TPA$^{+\cdot}$** to catalyst as shown in **Figure S17**. The contribution from **Ru^{II}P^{2+}** at this wavelength is subtracted as shown. The **TPA$^{+\cdot}$** signal in both **-Ru^{II}P(TPA)$^{2+}$** (blue) and **Ru^{II}P(TPA)(Cat)$^{2+}$** (purple) should follow the GSB kinetics at 470 nm as shown previously in **Figure S16**. As shown in the right panel, the kinetics of **TPA$^{+\cdot}$** in **Ru^{II}P(TPA)(Cat)$^{2+}$** could be described by:

$$[TPA](t)=-a_1 e^{-(k2+k8)t}+a_2 e^{-k3t}$$

where k_2, k_8 are determined by previous fitting in **Figure S16** and **S17**. The k_3 that corresponds to the **TPA$^{+\cdot}$** decay due to the hole transfer to the catalyst can be determined without interfering with the charge recombination at a later time. This is done by selectively investigating the kinetics between 1 ns and 50 ns shown in the right panel. Because without the catalyst (blue curve) the **TPA$^{+\cdot}$** shows no decay yet, it is safe to assume the decay at this time range is due to the transfer. This kinetics is fitted with an exponential to determine k_3, which is 3.6±1.6 ns.

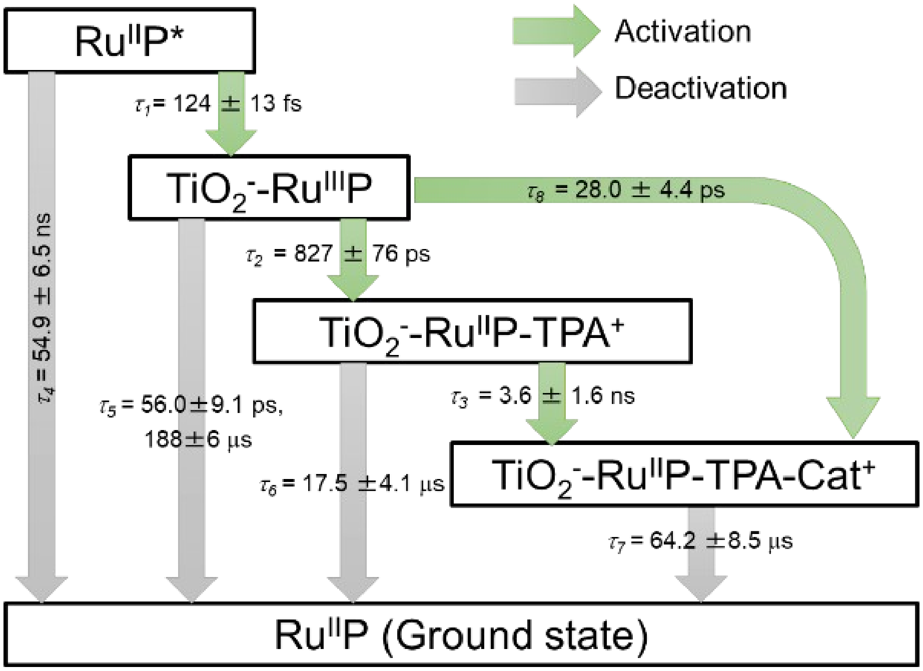

Table S3: The rate constants for electron transport processes shown in molecular assemblies were tabulated from the TA trace above.

1. Sherman, B. D.; Xie, Y.; Sheridan, M. V.; Wang, D.; Shaffer, D. W.; Meyer, T. J.; Concepcion, J. J., Light-Driven Water Splitting by a Covalently Linked Ruthenium-Based Chromophore–Catalyst Assembly. *ACS Energy Lett.* **2017,** *2* (1), 124-128.
2. Sampaio, R. N.; Troian-Gautier, L.; Meyer, G. J., A Charge-Separated State that Lives for Almost a Second at a Conductive Metal Oxide Interface. *Angew. Chem.* **2018,** *57* (47), 15390-15394.
3. Bangle, R. E.; Schneider, J.; Piechota, E. J.; Troian-Gautier, L.; Meyer, G. J., Electron Transfer Reorganization Energies in the Electrode–Electrolyte Double Layer. *J. Am. Chem. Soc.* **2020,** *142* (2), 674-679.
4. Wang, D.; Sampaio, R. N.; Troian-Gautier, L.; Marquard, S. L.; Farnum, B. H.; Sherman, B. D.; Sheridan, M. V.; Dares, C. J.; Meyer, G. J.; Meyer, T. J., Molecular Photoelectrode for Water Oxidation Inspired by Photosystem II. *J. Am. Chem. Soc.* **2019,** *141* (19), 7926-7933.
5. Wang, D.; Marquard, S. L.; Troian-Gautier, L.; Sheridan, M. V.; Sherman, B. D.; Wang, Y.; Eberhart, M. S.; Farnum, B. H.; Dares, C. J.; Meyer, T. J., Interfacial Deposition of Ru(II) Bipyridine-Dicarboxylate Complexes by Ligand Substitution for Applications in Water Oxidation Catalysis. *J. Am. Chem. Soc.* **2018,** *140* (2), 719-726.
6. Farnum, B. H.; Wee, K.-R.; Meyer, T. J., Self-assembled molecular p/n junctions for applications in dye-sensitized solar energy conversion. *Nat. Chem.* **2016,** *8* (9), 845-852.
